# Capturing the Data: Nutrition Risk Screening of Adults in Hospital

**DOI:** 10.3390/nu2040438

**Published:** 2010-04-01

**Authors:** Elizabeth Frew, Robyn Cant, Jennifer Sequeira

**Affiliations:** 1 Manager, Nutrition and Dietetics, Dandenong Hospital, PO Box 478 Dandenong Victoria, 3175 Australia; 2 Research Fellow, School of Nursing and Midwifery, Monash University, Churchill Victoria 3842 Australia; Email: Robyn.Cant@med.monash.edu.au; 3 Nutrition Technician, Nutrition and Dietetics, Dandenong Hospital, PO Box 478 Dandenong Victoria, 3175 Australia; Email: Jennifer.Sequeira@southernhealth.org.au

**Keywords:** adults, evidence translation, hospitals, malnutrition screening, Malnutrition Screening Tool

## Abstract

This study aims to explore limitations with the Malnutrition Screening Tool in identifyingmalnutrition risk, in a cohort of 3,033 adult Australian medical and surgical hospital inpatients. Seventy-two percent of patients were screened; illness and medical care limited access to others. Malnutrition risk (16.5%; n = 501) was found in all age groups with a trend to higher risk in medical wards; 10% (n = 300) of patients with communication barriers were excluded. Systematic screening increased dietitians’ referrals by 39%. Further research is required to enable screening of all patients, including those with communication issues with an easy to use valid tool.

## 1. Introduction

Nutrient demand is exacerbated in the case of illness [1]. In developed countries, a considerable proportion of patients in hospital are undernourished [2] and malnutrition is recognized as the most common condition occurring in hospitalized patients [3]. This condition afflicts between 20-50% of adults in hospital [4,5], co-existing with other disease processes [4]. Malnutrition is characterized by a protein/energy depletion [6]. Various descriptions of malnutrition are shown in Table 1. There are clear correlations between parameters reflecting poor nutrition, such as prealbumin or body mass index and rate of in-hospital complications, readmissions and mortality [3,8]. Malnourished patients recover more slowly from illness and experience more complications such as poor wound healing or altered immune function [7,9].

**Table 1:** Definitions of malnutrition.

*Author*	*Definition*
**Australian Government: Diseases Tabular (AN-DRG 10)^ a^**	In adults, BMI < 18.5 kg/m² or unintentional loss of weight (5%) with evidence of suboptimal intake resulting in moderate loss of subcutaneous fat and/or moderate muscle wasting.
**American Society for Parenteral and Enteral Nutrition ^b^**	Any disorder of nutrition status, including disorders resulting from deficiency of nutrient intake, impaired metabolism, or over-nutrition.
**World Health Organisation^c^**	Adults: Classification of Body Mass Index: < 18.49 kg/m^2 ^using reference charts for the relevant population.

**^a ^**National Centre for Classification in Health (NCCH). *The International Statistical Classification of Diseases and Related Health Problems, Tenth Revision, Australian Modification (ICD-10-AM) Tabular List of Diseases and Alphabetic Index of Diseases.* [6th Edition] Sydney: NCCH, The University of Sydney, 2008: p. 95. **^b^** Anonymous. Nutrition assessment- adults. *J. Parent Ent. Nutr. *2002, 26 (1), 9SA. **^c ^**World Health Organisation (WHO). *Management of Severe Malnutrition- a Manual for Physicians and Other Senior Health Workers.* Geneva, WHO, 1999: p 38.

Nutrition screening, or classifying patients to identify those at risk of malnutrition is therefore important, so that they can be referred for further assessment and, upon diagnosis of malnutrition, receive treatment [10]. In the absence of screening programs in hospital, many cases of malnutrition are missed [11].

Best evidence recommends nutrition screening of all adult patients early in their hospital admission [10,12]. A recent evidence-based practice guideline endorsed by the Dietitians Association of Australia supports this view [13]. However, unlike in the UK [14] there is no universal screening standard, nor routine screening in Australian hospitals [15]. A systematic process of screening is a precursor to more detailed assessment of those found to be at risk to determine whether malnutrition is present. Dietitians often employ the Subjective Global Assessment tool to establish presence or absence of malnutrition [16]. The malnutrition risk in a study of 5,149 Australian hospital patients amounted to 20% [17] and in 5,089 patients in UK hospitals, 28% [2]; suggesting there are large implications for hospital resources to enable both screening of newly admitted patients and also necessary treatments.

**Figure 1:**
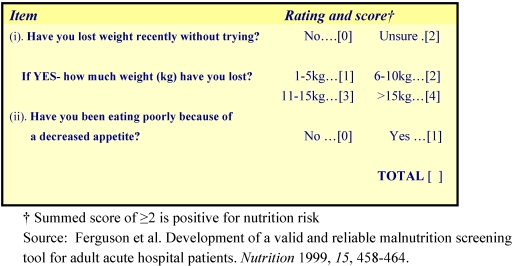
Malnutrition Screening Tool.

A number of tools have been developed which can reliably identify nutrition risk or poor nutritional status by measuring “characteristics known to be associated with nutritional problems” [18: p 13]. Each of these tools is based on multiple measures: either objective criteria such as body weight, body mass index (BMI) or other anthropometric and/or biochemical indices, or else subjective criteria such as reported weight loss and reported appetite changes. The Malnutrition Screening Tool (MST) is a valid instrument that identifies individuals who are at risk of malnutrition [19]. It uses subjective data and consists of two questions which ask about weight loss history and recent appetite, and can be completed by either a patient or healthcare worker. A total score of two or more indicates a positive score and risk of malnutrition (Figure 1).

This tool was validated in a private hospital population resulting in a sensitivity of 93% in identifying patients at nutrition risk, with specificity of 93%. Nutrition screening tools need to be easy to use and fast to complete when screening a large number of patients. The MST is recommended for ease of applicability and was confirmed as valid in the acute care setting in a randomized controlled trial. However, it is does not provide an avenue for screening patients who are unable to communicate, other than by use of the ‘Unsure’ rating [20]. This tool was used in the current study.

This paper reports on a cohort of adult patients in a study which evaluated nutrition screening operations in six wards of a large metropolitan public general hospital. The association of age and gender with nutrition risk will be explored and the implications of nutrition screening for patients’ nutrition management in hospital and for staff resourcing will be described.

### 1.1. Methods

The research setting was a large metropolitan acute public hospital in Melbourne, Australia. The MST was used to screen adult patients admitted to six medical and surgical wards over a period of five months (February to June) in 2008. Screening was undertaken by nutrition technicians who were trained by dietitians in use of the MST.

All consecutive new patients in the targeted wards were selected for inclusion. Patient admissions were identified from the electronic patient information-management system. Morning and afternoon ward rounds were conducted to screen new patients and screening was completed within 48 hours of admission. To reduce any duplication of assessments for patients, those already referred to a dietitian either by a doctor, an allied health professional or from a ward meeting were excluded from screening. Patients discharged the same day, or whose life expectancy was limited to days or hours, were excluded. For any screened patient unable to communicate a response to interview questions due to their condition, conscious state or English language difficulties (and where there was no alternative source of information such as from a family member), then the ‘unsure’ rating was allocated. Data on patients’ demography and screening outcome were recorded. Patients identified as at nutrition risk were referred to dietitians for assessment and nutritional diagnosis. Data on the number of patients referred to the dietitian were compared with the referral rate over the same months the previous year.

The project was approved by the ethics committee of the health service as a quality assurance project and so the formal consent of patients was not required.

### 1.2. Analysis

Statistical analysis was conducted using SPSS version 15 (Chicago, Il; SPSS Incorporated 2007). Descriptive statistics were used to compare response distributions and Chi-square and Spearman’s Rank Correlation Coefficient were used to examine between-group relationships. *P* = < 0.05 was regarded as significant.

## 2. Results

Of 4,216 patients admitted to six wards over five months, 72% (n = 3,033) were screened for malnutrition risk. Five hundred and one patients (16.5%) who responded to the interview questions were found to be at risk of malnutrition with a score of 2 or more. A further 305 (10%) were screened but were unable to answer interview questions because of either their medical condition which limited communication, or difficulties with the English language. They were thus rated using the ‘unsure’ category in the tool (score = 2). This scoring followed the MST protocol [19]. A hierarchy of reasons for this rating being given is listed in Table 2 in descending order of their frequency. Although this score technically deemed them ‘at risk’, the prevalence data was not included in the cohort of at-risk patients for the purposes of this report because the screening process of score allocation includes false positives and further steps are needed to specify their actual nutritional status.

A further proportion of admitted patients were excluded because their nutrition screening or assessment was already being conducted by a dietitian (an earlier referral being received from a treating health professional, at, or even prior to their admission). In all, 18% (n = 1,183) patients were not given a rating and were excluded altogether because they either did not meet the inclusion criteria, or were inaccessible due to medical examinations, surgery, procedures, other assessments, tests, treatments, palliative care, death, discharge home or else transfers to other wards or services. Furthermore, the currency of admission data- the listing of new patients- was one issue that might also limit accuracy of numbers for screening as well as patient transfer between wards.

**Table 2:** Hierarchy of reasons for screened patients being rated in ‘unsure’ MST category†

*Reason for inability to rate patients’ interview response*
**Uncertain interview response**: (i) patient uncertain or (ii) reports no recent loss of weight or appetite but on observation, appears underweight.†
**Limited skills in English language; **with no alternative information source
**Confusion or lowered cognitive state; **delirium, dementia, unconscious
**Severe disease state; **severely ill, unable to answer
**Hearing impairment; **lack of hearing appliance.

†Unsure rating triggers further assessment of nutritional status.

**Figure 2:**
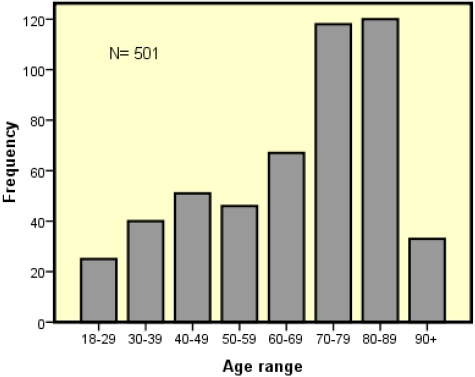
Age distribution of patients at malnutrition risk (MST-positive score by interview).

Of the 501 patients deemed at-risk, 45% (n = 224) were male and 55% (n = 277) were female. The age of patients at risk ranged from 18 to 102 years (mean 67 years; SD = 25). The most commonly occurring age of patients at risk was 86 years. There was no significant correlation between malnutrition risk and age or sex, although a trend was noted towards greater risk in females and in patients of older age. Patients aged 70-102 years comprised half (54%) of those found at risk as shown in Figure 2. However, it was also noted that patients in the 80+ age group were those who were often unable to be screened through interview communications and so the statistic may underestimate risk in this group. This data on age reflected the older age of patients in medical wards, for the mean age was 84 years (Southern Health, unpublished data 2009). Few patients in their ninth decade were found at-risk.

Almost twice as many surgical patients were screened compared with medical patients (Table 3). There were differences in risk results by ward category (medical; surgical; medical/surgical-orthopaedic) which did not reach a level of significance. A large proportion of patients screened and found at-risk were in medical wards: 22.5% of the medical patients, and 14% of surgical patients. The proportion of *all* patients found at risk was low in the medical/surgical (orthopaedic) ward (8%).

**Table 3:** Malnutrition risk by MST score ≥2 by hospital ward category†

*Ward category*	*Number screened*	*Patients at risk of malnutrition n (%)*	*Proportion of all those at risk %*
**Medical**	924	205 (22.5%)	41%
**Surgical**	1,780	255 (14%)	51%
**Med/Surg (orthopaedic)**	329	41 (12%)	8%
**TOTAL**	3,033	501	100%

†Patients screened by interview using MST, ‘unsure’ category excluded.

There was a significant positive correlation between older age ≥ 60 years and malnutrition risk based on various ward categories (r = 0.244; *p* < 0.01) as 85% of orthopaedic patients at risk were over 60 years versus 77% of at-risk medical ward patients and 56% of at-risk surgical patients. However, age accounted for only a small fraction of this variance (6%; r ^2^ =5.95).

Of 501 patients at risk, nearly all (92%) were rated at the lower end of the score range; MST score of 2 or 3. Half (52%) were rated score 2; 41% score 3 and for both score 4 and 5, 4%. 

Referrals to dietitians increased by 39% (1,593 compared to 1,143 the previous year). with the introduction of systematic nutrition screening compared with the same wards in the same months the previous year. All these results serve to illustrate differences in the cohort of patients which were identified once a systematic screening process was instituted.

## 3. Discussion

Over sixteen percent of 3,033 patients screened were found at risk of malnutrition. This proportion is less than would be expected in the whole hospital population because of the exclusion criteria which were applied to patients. However, the result is comparable with other studies of malnutrition risk which reported 20% risk rate in hospital patients in Australia [17] and 28% in the UK [2] using a large sample size. MST was selected as the nutrition risk screening tool due to it’s suitability for the hospital population [13] and ease of application with use of subjective data, not requiring each patient’s weight or anthropometry to be recorded. Van Venrooij et al concurred with the tool’s ease of use and found it quick and easy to use in a hospital population [20]. However, there were various barriers to recording data on every patient in the current study.

### 3.1. Barriers to Evidence Translation

Evidence translation is a key focus of care pathways for improving the quality of health care and positively influencing patient outcomes [10]. However, difficulties in getting the evidence into practice are highlighted by the results of this study. Routine nutrition screening of the adult patient population in acute hospital wards is based on implementation of best evidence [10,13], yet the process amounted to capturing data on only approximately three of every four admitted patients in medical and surgical wards. This was despite the efforts of nutrition technicians dedicated to the screening of patients on admission in six wards, over a period of five months.

Access to patients was limited by the characteristics of illness in patients and initiation of hospital procedures to treat them as they went through the trajectory of hospital admission, investigations and medical management. This raises the notion that, firstly, subjective assessments using instruments that require communication from a patient may be less useful than other objective tools for nutrition screening that are based on anthropometry and other indices that may be collected without patients’ communications. Secondly, that data might need to be collected from a patient’s nursing or medical assessment during the admission process- especially in the case of the 80+ age group. For example, the Malnutrition Universal Screening Tool (MUST) is recommended for assessment of hospital patients and can be implemented by weighing a patient or taking other body or skin-fold anthropometry, or making subjective estimations of BMI status [21]. However, no tool may be easy to use for all, or be universally applied in practice. A British study of 150 consecutive elderly hospital patients found that 23% could not be weighed nor provide screening information about weight history [22]. Alternatively, though, the MUST was applied to all of 328 patients as a screening tool for medical, surgical, orthopaedic and critical care in-patients in an acute hospital in England [23]. Excluded patients were those who were discharged or very recently admitted: so use of substituted subjective criteria may be useful in assessments. However, whether medical or nursing staff with direct responsibility for patient care may be better placed to screen for patients’ nutrition status is uncertain as a number of studies have shown that their screening rates are also deficient [14,24]. Many studies exclude a proportion of patients, not including the most dependent in hospital [8,9,19,25,26] and thus fail to portray the whole picture.

### 3.2. Age and Nutrition Risk

Older age is widely reported as a risk factor for malnutrition [2,8,9]. Nutrition risk in this study was related to advancing age in hospital patients, as two of every three patients at nutrition risk (68%) were aged over 60 years. Hospital admission data shows that the number of individuals over 65 years in public hospitals in the state of Victoria for multi-day stays is increasing annually [27]; that they comprise 40% of admissions and 70-90 year-olds account for more admissions than any other age group [27]. However, it should be noted that few patients in their ninth decade or older were found at-risk in this study. The reason may be related to a lower number of admitted patients in this age group because this is consistent with admission data [27]. Also it is possible these older patients are more difficult to screen because of increased dementia, delirium, depression or severity of illness. These factors suggest that screening the inpatient population is justified, both for those who can be interviewed and those who cannot. Arrangements need to be made to enable higher rates of screening, particularly inclusive of older patients. The effects of under-nutrition in the aged are well documented [1,28,29] and treatment outcomes can be positive. Once malnutrition is diagnosed, many of the biological and sociological factors that adversely impact upon their nutrition can be reversed [30] either in hospital [31,32] or after discharge. Continuation of interventions after discharge is important as home-based interventions were shown to be effective in decreasing nutrition risk [33]. A first step is to enable a patient’s risk identification through effective nutrition screening on admission to hospital.

However, nutrition risk is not confined to older age alone. Thirty-two percent of patients at risk in the current study were less than 60 years of age and 13% were aged less than 40. This data may be skewed because the hospital population under examination comprises many more elderly than young individuals. However, the overall results of the current study are wholly supported by those of a larger study of 5,089 hospital patients screened in 150 hospitals in the UK in 2008 [2]. This showed the age range of at-risk patients to be 18-103 years; more women at risk than men (30% *vs.* 26%); significantly higher risk in medical than surgical or orthopaedic/trauma patients (36% *vs.* 25% *vs.* 19%) and a significantly lower rate of malnutrition risk in hospital patients under 65 years than over 65 (23% *vs.* 32%) [2]. Rates of malnutrition diagnosed in a study of 1,882 hospital patients in Germany also indicate that all age groups (18 to 80+) are afflicted; with 15% prevalence of malnutrition in the 30-40 age group and 8% in the 18-30 age group when assessed using SGA [4]. Few studies have fully reported the incidence of risk in these younger patients (most often reporting the mean age) and this impact may therefore have been underestimated. When extrapolated to account annual hospital admissions, malnutrition risk, even in this younger group may have significant impact on hospital resources in managing treatments.

### 3.3. Workforce Implications

Studies indicate that malnutrition is under-recognized and under-treated [11,13] and screening is one way of ensuring a systematic process of timely referral to manage malnourished patients [13]. However, due to a shortened length of stay in hospitals [27], consequent increase in admissions, and aging of admitted patients [27], there is increasing pressure for nutrition technicians, dietitians and even nurses and doctors to manage the nutrition care of more patients even more rapidly. It is difficult to process patients in a timely manner because they are frequently moved to another ward or transferred elsewhere. Referrals to dietitians for assessment of patients increased 39% with routine screening. In the current study, older age patients with medical conditions in medical wards comprised a greater proportion of patients at risk and this concurs with other available data [2,5]. Dietitians may need to focus their staff numbers on work in medical wards to provide optimal services. Alternatively, however, the at-risk surgical patients were younger as 44% were aged less than 60 years and so these individuals may have potential for greater nutritional progress. Improvements in nutrient intake can be achieved in hospital and have been associated with reduced length of stay [34]. Other avenues of investigation such as particular diagnosis groups should be explored, because, for example, patients with cancer exhibit higher malnutrition risk than others [2,35]. However, research evidence is lacking about studies that demonstrate a positive impact of nutrition interventions upon the most important factor- patient outcomes. This includes rates of in-hospital complications as well as length of hospital stay and annual survival. Given this lack of data, these results present a dilemma for health administrators regarding how best to implement evidence-based practices and where to focus efforts to improve patients’ nutritional status.

### 3.4. Study Limitations

This study has limitations which influence whether results are applicable to other populations. Owing to the use of interview technique and exclusion criteria, the study was limited to a subgroup of hospital inpatients and therefore, the rates of nutrition risk may be lower than that in other studies. Patients’ treatments, their condition and hospital work practices limited access to a greater proportion of admitted patients, so that characteristics of patients who were not screened and also accurate prevalence data are unknown. Potential assessment bias was limited by the training of nutrition technicians who conducted the screening. Even though an extensive sample was included, care must be taken in interpreting the results as the sample may not be representative of the whole hospital population. Nevertheless, the results raise issues about the screening of patients on admission to hospital to identify risk, with the aim of increasing understanding to help enable a more effective screening process to capture data on all the relevant hospital population. While we are currently using the MST, we are investigating new developments to more accurately identify all acute patients at risk.

## 4. Conclusions

Malnutrition risk consequent to nutrient deficiency was identified in a cohort of hospital patients in a metropolitan acute care public hospital by nutrition technician staff. Three of every five medical and surgical patients were able to be screened, the screening of others being limited by the nature of patients’ illnesses and their hospital care. Nutrition risk was related to advancing age, although patients of all ages were at risk. There may be benefit in using qualitative screening tools such as MST for patients who can communicate. Further research is warranted to enable a more effective screening process and further development of tools that will capture nutrition screening data on all targeted patients in the hospital population.
